# Neutralization Capacity of Monovalant Antivenom Against Existing Lethal Scorpions in the Turkish Scorpiofauna

**Published:** 2017

**Authors:** Ozcan Ozkan, Ersen Aydın Yağmur

**Affiliations:** a *Department of Biology, Çankırı Karatekin University, Çankırı, Turkey.*; b *Alaşehir Vocational School, Celal Bayar University, Alaşehir, Manisa, Turkey.*

**Keywords:** Turkish Scorpiofauna, lethality, *Androctonus crassicauda* antivenom

## Abstract

In this study, *Mesobuthus gibbosus* and *Mesobuthus eupeus eupeus* venom samples were compared for lethality, *in-vivo* effects and proteins. Neutralization capacity of monovalent *Androctonus crassicauda* antivenom (RSHA anti-Ac) was tested against the lethal effects of the venoms.

Venom was obtained from mature scorpions by electrical stimulation of the telson. The lethality of the venom and potency of Horse RSHA anti-Ac were determined in Swiss mice. The protein profiles of the scorpion venoms were analysed by NuPAGE® 4–12% gradient Bis-Tris gel followed by Coomassie blue staining. Western blotting was performed to determine immunogenic compounds in the venom samples.

The median lethal doses of *M. e. eupeus, M.gibbosus* scorpion and *A.crassicauda* venoms were determined to be 1.92 mg/kg by i.v. injection route, 0.67 mg/kg and 0.24 mg/kg by s.c. injection route, respectively. *A.crassicauda *(Olivier, 1807) venom was used as control. One millilitre of the RSHA anti-Ac neutralises 23 LD_50_ of *M. e. eupeus*, 32 LD_50_ of *M.gibbosus* and 42 LD_50_ of *A. crassicauda* venom in mice. Analysis of electrophoresis indicates that three scorpion venoms posses low molecular weight proteins. Immunoblotting indicated that RSHA anti-Ac strongly reacted with both the specific venom and *Mesobuthus *species venoms which have antigenic similarity.

The result of our study showed that* M.e. eupeus *and* M.gibbosus* could be medically important scorpions for humans, particullary children. The RSHA anti-Ac can be used in the treatment of envenomation by *M. e.eupeus* and *M.gibbosus* scorpion stings.

## Introduction

Envenomation by arachnids causes significant injuries all over the world (1). Scorpion sting is the most important type of arachnid envenomation resulting in adult morbidity and pediatric mortality (2). Lethal scorpions mostly belong to the Buthidae family (3), among which, species of the genera *Androctonus*, *Leiurus*, and *Mesobuthus *are mainly responsible for envenomation in Turkey (4). 

Although *Mesobuthus eupeus* has been considered to be a single species for many years, two subspecies of *M. eupeus*, which have been identified as *M. eupeus phillipsii* and *M. eupeus eupeus *have been recorded in the Turkish scorpiofauna recently by Kovařík *et al.* (5). Subsequently, Mirshamsi *et al.* (6) revised subspecies of *M. eupeus* existing in Iran that include the subspecies *M. eupeus phillipsii* and *M. eupeus eupeus*, and *M. eupeus phillipsii* elevated to species level as *M. phillipsii*. 

*Mesobuthus gibbosus* occurs throughout Anatolia, except for the Black Sea coast in northern Turkey, European Turkey, Eastern and South-eatern Anatolia, and is the most abundant scorpion in this region (7-10). *Mesobuthus* is one of the most widely distributed genera of the family Buthidae, with species being present throughout Turkey. Stings by *Mesobuthus *species are more frequent than those by other scorpion species (4, 11-13). The previous reports also indicated that those *Mesobuthus* species are involved in numerous serious and deadly envenomations, especially in children (14–18). 

Scorpion anti-venom is the only specific treatment for scorpion envenomation and is still widely used in many countries (19, 20) as there are no vaccines or other effective agents against animal venoms (21). However, anti-venoms are still administered empirically and consequently their efficacy is controversial, particularly in the case of mild or moderate scorpion envenomations (22). As venom is a complex mixture of antigens wherein not all components are equally important for the production of neutralizing antibodies, the identification of immunogenic protein(s) and/or their neutralizing epitopes may lead to the use of more clearly defined substances as immunogens to develop efficient anti-venoms. RSHA anti-Ac has been produced at the Refik Saydam Public Health Agency (RSHA), Ankara in Turkey since 1942 by venom of *A. crassicauda* and administering to horses (23, 24). Consequently, identification of the factors that lead to effective antivenom generation is important. With the median lethal doses of* M.gibbosus* (12), *M.eupeus (*25) scorpion venom and the potency of RSHA antivenom previously determined, there was no confirmed cross-reactivity of the three venoms with the horse anti-venom produced by RSHA, Turkey. We studied venom samples obtained from two different *Mesobuthus* which were *Mesobuthus gibbosus *from the West of Turkey and *M.eupeus eupeus* from Southeastern Turkey.

The present study is aimed to determine the paraspecific effects and potency of RSHA anti-Ac against the venom and determine the protein profiles (molecular weight), minimal lethal dose and also to show *in-vivo* effects in mice to improve quality and paraspecific activity of national antivenom.

## Experimental

*Scorpions*

**Figure 1:**
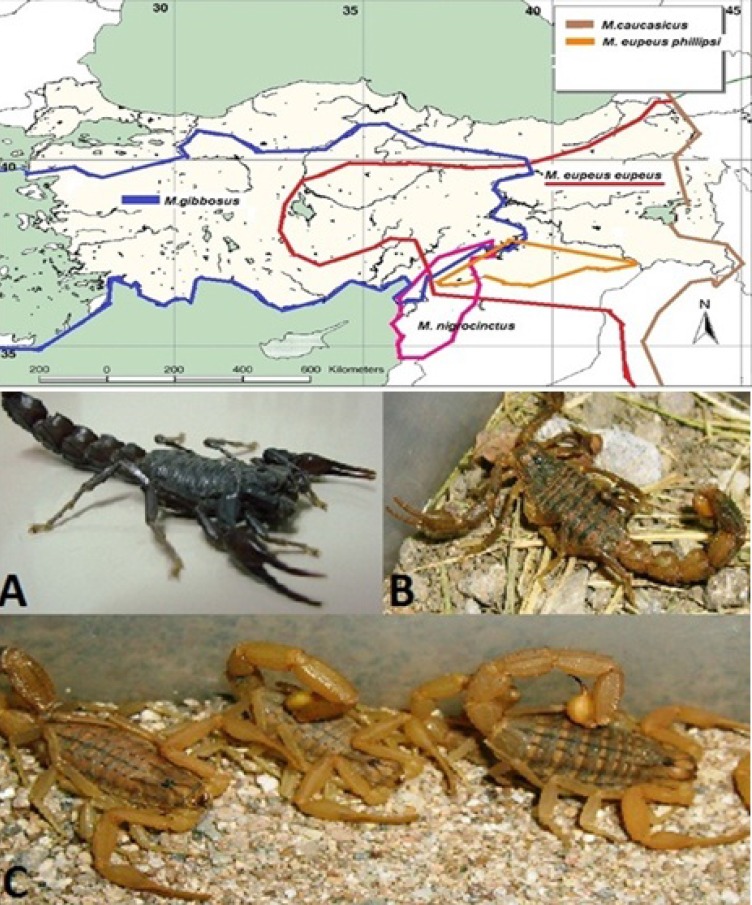
Map of distribution of Mesobuthus speciesin Turkey (Blue line; *Mesobuthus gibbosus, *red line; *Mesobuthus eupeus eupeus, *orange line: *M. e. phillipsii,* brown line: *M.* *caucasicus, *green line: *M.* *nigrocinctus* )*. *The venom of *A. crassicauda* (**A**) has been used in the production of antivenom. *M. e. phillipsii *was commonly found in the Southeast Anatolia Region, but *M.eupeus eupeus *(**B**) was reported only in the Karacadag Mountain (Red circle) within the region of Turkey (Kovařík, et al., 2011)*. Mesobuthus gibbosus *(**C**) is the source of many scorpion envenomation cases in the Aegean, Mediterranean, Marmara and Central Anatolia regions of Turkey

*M.gibbosus* was collected from Aydın, *M.e.eupeus* from Karacadağ Mountain (Şanlıurfa, 37°44› 45»N 39°49›50»E, 1752 m) and *A.crassicauda* from Şanlıurfa province at night, by using a UV lamp (Figure 1). Avoiding cannibalism, captive scorpions were housed in individual boxes at the Department of Entomology, Faculty of Veterinary Medicine, Ankara University, Turkey. The scorpions were fed with crickets or cockroaches and received water daily.

*Venoms*

Venom was obtained from mature *M. eupeus eupeus*, *A. crassicauda* scorpions (Figure 1) from Şanlıurfa province and *M. gibbosus *scorpions from Aydın province by electrical stimulation of the telson (26). The venoms were dissolved in sterile double-distilled water and centrifuged at 14,000 rpm for 15 min at 4 ºC. The supernatant was stored at -20^o^C. The protein content of the venoms was determined by the Bradford method using BSA as the standard (27). 

*Antivenom*

Horse RSHA anti-Ac, produced by the Refik Saydam Public Health Agency is the unique antivenom available in the country and has been used to treat all scorpion stings in the country since 1942 (4). Dose is normalized to neutralize two minimal lethal doses (MLD) in rats when they are tested subcutaneously (4).

*Experimental animals*

Experimental protocols followed the guidelines of the Bioethics Commission, the RSHA, and were approved by this Commission. *Swiss* mice of both sexes were employed to determine the median lethal dose (LD_50_) and the median effective dose (ED_50_) by intravenous (IV) and subcutaneous (SC) route of administration. They were breed in animal facility of the RSPHA. The animals were housed under controlled temperature (20±2°C), with a 12:12 light/dark schedule and were fed with commercial rodent pellets and water *ad libitum* throughout the experiment.

**Figure 2:**
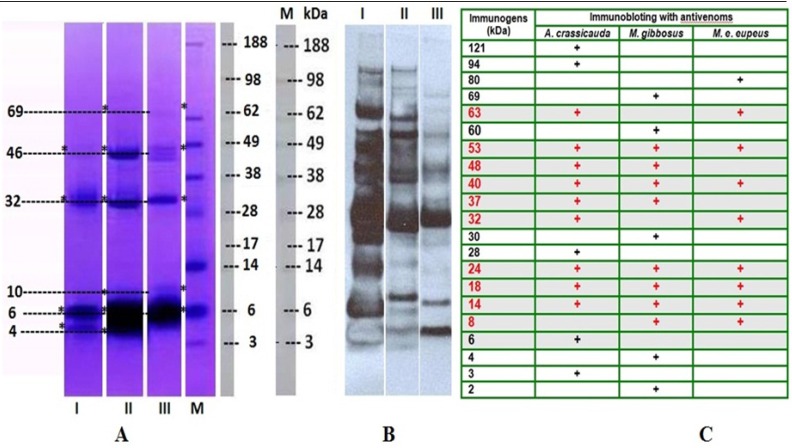
(A) The proteins of the venom of *M. e. eupeus* (Lane I), *M. gibbosus *(Lane II) and *A. crassicauda* (Lane III) were separated by using 4%-12% NuPAGE gradient gel electrophoresis. (B) Immunoblotting was carried out to evaluate the reactivity of the scorpion venoms components against anti-*A. crassicauda* horse antivenom (1: 4000), and the immunogenic compounds in both venom samples were determined. *A. crassicauda* venom was used as control. Lane M: Molecular weight markers –188 KDa Myosin, 98 KDa Phosphorylase, 62 KDa BSA, 49 KDa Glutamic Dehydrogenase, 38 KDa Alcohol Dehydrogenase, 28 KDa Carbonic Anhydrase, 17 KDa Myoglobin Red, 14 KDa Lysozyme, 6 KDa Aprotinin, 3 KDa Insulin, B Chain (SeeBlue® Plus2 Pre-Stain). (C) After immunoblotting, molecular weights of immunogenic proteins in all venom samples were calculated with Molecular Imaging Software

*Lethal potency of the venoms*

The lethality of the venom was determined as described by Behrens and Karber (28) using *Swiss* mice. For per each dose group, five mice were injected with increasing volume doses of *M. eupeus eupeus* venoms (IV injection: 28.6, 30.1, 33.2, 35.4 µg / 16g mouse [n: 20 mice]), *M. gibbosus* venoms (SC injection: 7.6, 11.3, 14.9, 18.5 and 22.1 µg / 20g mouse [n: 25 mice]) and *A. crassicauda* venom (SC injection; 3.15, 3.49, 3.89, and 4.14µg/ 20g mouse [n: 20 mice]) diluted in physiological saline solution (PSS: 0.85% NaCl). An equivalent volume of PSS was injected into five mice as negative control group. Deaths occurring after 24 h were recorded in order to determine the median lethal dose. The lethalty was expressed as the LD_50_.

*Antivenom potency assay*

Neutralization studies were carried out by mixing a constant fixed amount of RSHA anti-Ac (1 mL) with various dilutions of *M. eupeus eupeus* venoms (IV injection: 10, 14, 18, 25 and 28LD_50 _[n: 30 mice]), *M. gibbosus* venoms (SC injection; 29, 31, 33, 35, 38 and 40LD_50 _[n: 36 mice]) and *A. crassicauda* venoms (SC injection; 10, 20, 30, 40 and 50LD_50 _[n: 30 mice]) and the mixture was incubated for 30 min at 37 °C. In the control groups, mice (each group n: 2) were injected with 2 LD_50,_ each of venom dissolved in PSS without RSHA anti-Ac. The numbers of surviving mice were recorded up to 48h. Neutralization capacity of RSHA anti-Ac was expressed as ED_50_, which corresponds to 1 mL RSHA anti-Ac in which the activity of the venom was reduced by 50%. 

*Assay of the envenomation*

The mice injected with the scorpion venoms were used for lethal potency determination and they are observed for the assessment of their symptoms from the moment of the injection until the end of experiment. 

*Gel electrophoresis of the venoms*

Polyacrylamide gel electrophoresis of venom sample was carried out by following LaemmLi (29) method. For separation of proteins, samples were run on NuPAGE^®^ Novex^®^ 4-12% gradient Bis-Tris gel (Invitrogen) in MES SDS Running Buffer (Invitrogen - 50mM MES, 50mM Tris-HCl, 1% SDS, 1.025mM EDTA) using Xcell SureLock Mini Cell (Invitrogen) by following standard manufacturer protocol. SeeBlue^®^ Plus2 Pre-Stained Standard (Invitrogen) was run in parallel in order to calculate the molecular weights of proteins. Detection of proteins was carried out initially by Coomassie blue staining. The gel was then scanned and molecular weights of the proteins were calculated with Molecular Imaging Software (Kodak MI).

*Immunoblotting*

Western blotting was performed to determine immunogenic compounds in the venom samples. Scorpion venoms of *Androctonus crassicauda* (15µg), *M.gibbosus* (15µg), and *M. eupeus eupeus *(20µg), were applied to NuPAGE Novex 4–12% Bis–Tris gradient gel (Invitrogen). Proteins were transferred onto iBlot gel transfer stacks polyvinylidene fluoride (PVDF) membrane (Invitrogen) using the Blot dry blotting system (Invitrogen). Blots were then blocked with 5% skimmed milk powder in Tris-buffered saline (TBS, pH:7.5) containing 0.1% Tween20 (TBS-T) for 1 h at room temperature (RT). After the membrane was washed three times with TBS-T, it was incubated at RT with 1: 4000 dilution of the antivenom. Blots were washed and then incubated with horseradish peroxidase-conjugated anti-horse antibody and HRP- conjugated anti-rabbit (1: 3000) for 60 min at RT. PVDF membrane was again washed with TBS-T and antigens were visualized by using the Immun-Star HPR Chemiluminescent subtrate (BioRad). The membranes were exposed to X-ray film and developed in a darkroom.

## Results

The identity of the scorpions were confirmed as *A. crassicauda*, *M. gibbosus*, and *M. eupeus eupeus *(Figure 1) using a stereomicroscope.

*Lethal potency of the scorpion venom*

Protein content of *M. e. eupeus, M.gibbosus *and* A. crassicauda* scorpion venom was found to be 3.72, 1.15 and 1.54 µg protein/ µl, respectively. The median lethal dose for* M. eupeus eupeus, M.gibbosus *and* A. crassicauda* scorpion venoms was determined in mice. The LD_50_ of *M. eupeus eupeus *was determined as 1.92 mg/kg by i.v injection route. The LD_50_ of *M.gibbosus *and* A. crassicauda* scorpion venoms were found as 0.67 mg/kg and 0.24 mg/kg by s.c injection route, respectively.

*Assessment of the experimental envenomation after venom injection*

Similar symptoms were observed in mice during LD_50_ determination. After all venom injections, mice showed some symptoms such as mastication, mouth rubbing, squeaking and fight, restlessness, aggressive behavior, humpback, tremor, tachypnea, deep dyspnea, excessively hypersalivation and lacrymation, weakness, convulsions, paralysis, coma resulting in death. 

*The neutralizing capacity of Androctonus crassicauda antivenom*

To assess the efficacy of the monovalent antivenom, increasing doses of the venom were used while the amount of the antivenom (1 mL) was kept constant. Neutralization capacity of one mL antivenom was found to be against 23 LD_50_ of *M. e. eupeus*, 32 LD_50_ of *M.gibbosus* and 42 LD_50 _of *A. crassicauda* venoms while all the control mice died. 

*Determination of protein profiles*

The protein profiles of the scorpion venoms were analyzed by NuPAGE^®^ 4-12 % Bis-Tris gel, and followed by Coomassie blue staining. Proteins of the venoms were determined between 3 and 188 kDa by electrophoresis on gradient gel as shown in Figure 2-A. In the electrophoretic analysis, *M. eupeus eupeus* scorpion venom showed protein bands as ~ 4, 6, 32, and 46kDa (Figure 2-A; Lane I), and detected protein patterns of *M.gibbosus* scorpion venom were as ~ 4, 6, 10, 32, 46 and 69kDa (Figure 2-A; Lane II), and those of *A.crassicauda *scorpion venom as ~ 6, 10, 32, 46 and 69kDa (Figure 2-A; Lane III), after staining with Coomassie Blue. When they are compared, they showed only four similar protein bands ~ 4kDa, 6kDa, 32kDa and 46kDa (Figure 2-A) between the *Mesobuthus* species venoms. The common protein bands were also ~ 6, 32, 46kDa among three venom samples.

RSHA anti-Ac strongly showed reaction with both the specific venom and the other two *Mesobuthus* species venoms. As shown in Figure 2B, specific antibody showed reaction with the *M. e. eupeus* venom (3, 6, 14, 18, 24, 28, 32, 37, 40, 48, 53, 63, 94 and 121 kDa proteins), (Figure 2-B, Lane I), and with the* M. gibbosus* venom (4, 8, 14, 18, 24, 32, 40, 53, 63 and 80 kDa proteins), (Figure 2-B, Lane 2) and also with the *A.crassicauda* venom (2, 4, 8, 14, 18, 24, 30, 37, 40, 48, 53, 60 and 69 kDa proteins), (Figure 2-B, Lane 3). Immunoblotting indicated 20 proteins as immunogens in three venom samples. The common immunogen proteins were 8, 14, 18, 24, 40, and 53kDa between *M. e. eupeus* venom and *M. gibbosus* venom according to the result of immunoblotting. Comparison of western blotting profiles of all venom samples showed proteins with similar molecular weights 14, 18, 24, 40 and 53kDa (Figure 2-C).

## Discussion

*M. eupeus* is found in Armenia, Azerbaijan, Georgia, Iran, Russia, Turkey and Turkmenistan. *M. gibbosus* is known to be present in Albania, Bulgaria, Macedonia, Montenegro, Greece and Turkey (5, 10, 30, 31). Toxicity of the venom and the signs of the scorpion envenomation depend on major factors such as scorpion species, venom composition and the victim´s physiological reaction to the venom (4, 32).In the study carried out in 2005, a total of 24, 261 cases of scorpion sting were reported (13). The members of genus *Mesobuthus* is mostly responsible in these stings in the country (33). Analysis of data in the literature showed fatal cases, especially in children, reported from different provinces of Turkey, where distribition of both the *Mesobuthus* species ranges (14–18). In Asian countries, most of the victims had been affected by *M. eupeus* (34–37). Therefore, *M.eupeus* is one of the major species responsible for scorpion sting in Asian countries. Lebez *et al.* (38) found that toxicity of *M. gibbosus *was 0.4 mg for mice and 2.4 mg for rats. 

On the other hand, Özkan (39) obtained venom from macerated *M.gibbosus *telson to determine lethality reported 10 mg/kg lethal dose by s.c injection route. In current study, the median lethal dose of *M.gibbosus* scorpion venom was found to be 0.67 mg/kg by s.c. injection route. Our current study confirmed that *A. crassicauda* venom is three times more lethal than *M.gibbosus *venom as previously reported by Özkan *et al.* (12). Therefore, *M.gibbosus* is known as a potentially dangerous European scorpion (38). This study showed that *M.eupeus *and* M.gibbosus* could be medically important scorpions for humans and particularly children. 

Zayerzadeh *et al.* (40) reported that lethal dose of *M.eupeus *venom from Iran notified 4.5mg/kg and the venom injection in rabbits evoked severe pulmonary edema and death. Latifi and Tabatabai (41) found that LD50 of *M. eupeus* scorpion venom from Iran was 1.36 mg/kg while Hassan (42) found it to be 1.45 mg/kg by i.v injection route. Özkan and Carhan (25) determined that the median lethal dose for *M. eupeus* from the Central Anatolian region of Turkey to be 0.18 mg/kg by i.c.v. injection route. They stated that *M. eupeus* scorpion venom is 11 times less toxic than *A. crassicauda* scorpion venom. In our study, LD_50_ of *M. e. eupeus *was determined that to be 1.92 mg/kg by i.v. injection route. We found similar results among toxcitiy of *A. crassicauda *venom and *M.e. eupeus *venom. Mice, experimentally envenomed with *M. gibbosus* venom, manifested tremor, hypersalivation, mouth and nose bleeding, paralysis and death (39). In our study, similar sympathetic and parasympathetic signs except for mouth and nose bleeding were observed in mice during lethality determination,

All scorpion species with high potential of mammal specific neurotoxins belong to the Buthidae family, due to their neurotoxic effects and medical importance (2). Scorpion venoms can be classified into two groups according to their molecular weights, long-chain (6,500 to 7,800 Da) and short-chain neurotoxins (3,000 to 4,400 Da) (43-45). Uçar and Taş (46) indicated that protein bands’ molecular weight ranges from 6.5 to 210 kDa detected in the venom of *M. gibbosus* from Manisa province. In current work, analysis of electrophoresis shows that all scorpion venoms possess low molecular weigth proteins. There has been antigenic similarity among the venom of scorpions belonging to the Buthidae family (47). Ozkan *et al.* (48) reported that three protein bands with molecular masses of 70, 87 and 100 kDa were detected in *M. gibbosus *and* M. eupeus* scorpion venom samples, according to the SDS-PAGE analyses. Ozkan and Ciftci (33) indicated that the protein bands with molecular masses of 28, 30, 33, 68 and 98 kDa were detected in the venom of *M. gibbosus* from Mugla province. In the current study, western blotting showed that RSHA anti-Ac strongly reacted with *A.crassicauda*, *M. gibbosus *and* M. e. eupeus* scorpion venoms which have antigenic similarity (Five protein bands with molecular masses of 14, 18, 24, 40, 53 kDa). 

Altınkaynak *et al*. (14) recommended that the Turkish antivenom (*A.crassicauda* antivenom) is used to treat *M. gibbosus *envenomation cases, since it had been effective in 91.6% of the cases which were studied. The antivenom was not effective in only two cases (9 and 13-month-old infants) because the patients had late received the first aid and hospitalization, and the sting site was on the neck (14). Soker and Haspolat (49) verified that the Turkish antivenom was effective in 90.3% of all scorpion related cases in South-Eastern Anatolia. In many studies, it was reported that *A. crassicauda* antivenom was capable of neutralizing various scorpion venoms in mice (12, 23, 50, 51). In our study, bioassays showed similar paraspecific activity with previous potency studies. We demonstrated that 1 mL of the antivenom can neutralize 23 LD_50_ *M. e. eupeus,* 32 LD_50_ *M. gibbosus* and 42 LD_50_ *A. crassicauda* venom in mice.

## Conclusion

Lethal potency of the venoms indicated that *M.e.eupeus *and* M.gibbosus* could be dangerous for people, especially children. These scorpion venoms possess low molecular weight of proteins according to the analysis of electrophoresis. The western blot analysis also shows that three venoms have similar antigenicity pattern. RSHC anti-Ac (1 mL) was capable of neutralizing *M. e. eupeus* venom (23LD_50_), *M.gibbosus* venom (32LD_50_) and *A. crassicauda* venom (42LD_50_). The RSHC anti-Ac can be used in the treatment of envenomation by *M. e.eupeus* and *M.gibbosus* scorpion stings.
